# C-853-05. Gel Sheets Efficacy for Hypertrophic Burn Scars: A Randomized Controlled Trial

**DOI:** 10.1093/jbcr/irag033.073

**Published:** 2026-04-06

**Authors:** Stéphanie Jean, Bernadette Nedelec, Zoë Edger-Lacoursière, Valérie Calva, Jakub Sawicki, Elisabeth Marois-Pagé, Geneviève Schneider, Ingrid Malo-Leclerc, Danielle Shashoua, José A Correa

**Affiliations:** Université de Montréal, Montréal, QC; McGill University, Montréal, QC; Villa Medica Rehabilitation Hospital and McGill University, Montréal, QC; Hôpital de réadaptation Villa Medica, Montréal, QC; Centre de Recherche, Centre hospitalier de l'Université de Montréal, Longueuil, QC; Hôpital de réadaptation Villa Medica, Montréal, QC; Hôpital de réadaptation Villa Medica, Montréal, QC; Hôpital de réadaptation Villa Medica, Montréal, QC; Hôpital de réadaptation Villa Medica, Montréal, QC; McGill University, Montréal, QC

## Abstract

**Introduction:**

The use of gel sheets for the treatment of hypertrophic scars (HSc) was introduced in the 1980s and has since become a widely adopted intervention. However, the quality of evidence supporting their efficacy remains limited, with most studies focusing on post-surgical linear scars rather than hypertrophic burn scars. The objective of this study was to conduct an appropriately powered, evaluator-blinded, randomized controlled trial to assess the effect of gel sheets on established burn HSc, using patient-matched control scars receiving usual care.

**Methods:**

Thirty-six burn survivors with two anatomically independent scars were selected based on ultrasound-determined thickness (>2.034 mm). To ensure homogeneity, pre-treatment thickness measurements of both sites were within 0.5 mm, and an erythema index >300 was used to confirm the scars as immature HSc. Each pair of scars was randomly allocated to either the treatment or control group. The treatment scar received gel sheet therapy in addition to usual care, while the control scar received only usual care over a three-month period.Objective assessments of scar thickness, elasticity, erythema, transepidermal water loss (TEWL), and melanin content were performed at baseline, 1 month, 2 months, and 3 months of treatment, with a follow-up one month after cessation of treatment. Itch and pain were recorded using a visual analogue scale, and adherence to treatment was tracked monthly during follow-up appointments.

**Results:**

Analysis of covariance (ANCOVA), controlling for pre-treatment values, revealed no statistically significant differences between treated and control scars at the 3-month endpoint or the 1-month post-treatment follow-up across all measured parameters, including thickness, elasticity, erythema, TEWL, melanin content, and itch intensity. However, in the subgroup of participants who wore the gel sheets for more than 16 hours per day, ANCOVA revealed a significant increase in elasticity in the treated scars compared to control. Paired t-tests comparing all pre-treatment and 3-month post-treatment values showed significant reductions in thickness and TEWL, and increased elasticity in both treated and control scars. No significant changes were observed in erythema or melanin levels.

**Conclusions:**

In conclusion, scar thickness, elasticity, and TEWL improved over time in both treated and control sites. While no significant group differences were observed in the overall analysis, improved elasticity was found in treated scars among participants who adhered to wearing gel sheets for more than 16 hours daily.

**Applicability of Research to Practice:**

These findings suggest that gel sheets may only enhance scar outcomes when used consistently for extended periods.

**Funding for the study:**

Foundation funding.

